# Extracellular Vesicles Shedding Promotes Melanoma Growth in Response to Chemotherapy

**DOI:** 10.1038/s41598-019-50848-z

**Published:** 2019-10-09

**Authors:** Luciana Nogueira de Sousa Andrade, Andréia Hanada Otake, Silvia Guedes Braga Cardim, Felipe Ilelis da Silva, Mariana Mari Ikoma Sakamoto, Tatiane Katsue Furuya, Miyuki Uno, Fátima Solange Pasini, Roger Chammas

**Affiliations:** Centro de Investigação Translacional em Oncologia (LIM24), Instituto do Câncer do Estado de São Paulo, Hospital das Clinicas (HCFMUSP) Faculdade de Medicina da Universidade de São Paulo, Av. Dr. Arnaldo, 251, São Paulo, SP CEP 01246-000 Brazil

**Keywords:** Cancer, Cancer microenvironment

## Abstract

Extracellular vesicles (EVs) are emerging as key players in intercellular communication. EVs can transfer biological macromolecules to recipient cells, modulating various physiological and pathological processes. It has been shown that tumor cells secrete large amounts of EVs that can be taken up by malignant and stromal cells, dictating tumor progression. In this study, we investigated whether EVs secreted by melanoma cells in response to chemotherapy modulate tumor response to alkylating drugs. Our findings showed that human and murine melanoma cells secrete more EVs after treatment with temozolomide and cisplatin. We observed that EVs shed by melanoma cells after temozolomide treatment modify macrophage phenotype by skewing macrophage activation towards the M2 phenotype through upregulation of M2-marker genes. Moreover, these EVs were able to favor melanoma re-growth *in vivo*, which was accompanied by an increase in Arginase 1 and IL10 gene expression levels by stromal cells and an increase in genes related to DNA repair, cell survival and stemness in tumor cells. Taken together, this study suggests that EVs shed by tumor cells in response to chemotherapy promote tumor repopulation and treatment failure through cellular reprogramming in melanoma cells.

## Introduction

Horizontal transfer of biological information by extracellular vesicles (EVs) is an evolutionarily conserved mechanism for cellular communication from prokaryotes to eukaryotes^1^. EVs are spherical particles enclosed by a phospholipid layer with a diameter ranging from 30 nm up to 1 µm that are secreted by different cell types. These vesicles can be divided into different classes based on their biogenesis, origin and size. Exosomes (50–150 nm) have their origins in multi-vesicular bodies that fuse with plasma membrane and undergo exocytosis^2–4^. Microvesicles are more heterogeneous in size, ranging from >100 nm to 1 µM in diameter, and are plasma membrane-derived vesicles shed as result of the budding of plasma membrane^5^. Both classes of EVs are the most widely characterized vesicles and have been described as important mediators of intercellular communication in physiological and pathological conditions.

In the past few years, the role of EVs in cancer has been highlighted by several groups. In gliomas, it was shown that the mutated form of EGFRVIII is transferred via microvesicles to surrounding tumor cells lacking this receptor, conferring a growth advantage to these cells^6^. Using a normal human gastric epithelial organoid system, Ke *et al*.^7^ demonstrated that EVs derived from esophageal adenocarcinoma cells are able to induce a neoplastic phenotype in the non-transformed cells, indicating the role of these vesicles in the malignant transformation. Moreover, numerous studies showed that tumor-derived EVs are involved in the acquisition of chemoresistance. Breast cancer cells treated with paclitaxel secreted exosomes enriched with survivin, an anti-apoptotic protein. The authors observed that these exosomes promoted the survival of breast tumor cells to chemotherapy, contributing to chemoresistance in this model^8^. In glioblastoma, exosomes derived from tumor cells harboring PTPRZ1-MET fusion conferred temozolomide resistance to recipient GBM cells, which was independent of O-6-methylguanine-DNA-methyltransferase levels^9^. In prostate cancer, docetaxel resistance was also mediated by exosomes^10^. In A549 lung cancer cells, exosomes secretion under cisplatin treatment decreased the sensitivity of other A549 cells to this drug^11^, suggesting that EVs impair chemotherapy efficacy.

The pro-tumoral effects of EVs are not restricted only to malignant cells. The angiogenic role of these vesicles in tumor microenvironment has been described in different solid tumors. Nazarenko *et al*.^12^ using a rat adenocarcinoma model observed that tumor-derived exosomes induced endothelial cell proliferation, migration and sprouting, supporting their role in angiogenesis. Exosomes secreted by colorectal cancer cells under hypoxia promoted endothelial proliferation and migration *in vitro* and enhanced tumor growth and angiogenesis *in vivo*^13^. On the other hand, microvesicles enriched in miR-29a/c secreted by gastric carcinoma cells inhibit tumor growth and angiogenesis *in vivo*^14^. Besides that, the role of EVs in pre-metastatic niche (PMN) formation has gained attention in the last few years. Jung *et al*.^15^ demonstrated that both exosomes and soluble factors from rat adenocarcinoma cells mediate the formation of PMN in lungs and lymph nodes. Melanoma-derived exosomes promoted PMN formation through induction of vascular leakiness and bone marrow progenitor cells reprogramming towards a pro-vasculogenic phenotype^16^. In pancreatic cancer, tumor cells-derived exosomes were shown to promote the PMN in liver. In this model, exosomes are uptaken by Kupffer cells, leading to the secretion of TGF-β by these cells, which was followed by secretion of fibronectin by hepatic stellate cells and PMN organization^17^.

Regarding the immune system, although initial studies showed an immunosupressive role of EVs, it has been shown that these vesicles can exert both a pro and anti-tumoral effect which seems to be dependent on vesicle cargo and cell type. Focusing on macrophages, breast cancer cells-derived exosomes promoted tumor-associated macrophages (TAMs) activation through NFkB and pro-inflammatory cytokines release^18^. In gastric cancer, tumor cells-derived exosomes activated macrophages to secrete pro-inflammatory cytokines which promoted tumor cell proliferation and migration^19^. In addition, Baj-Krzyworzeka *et al*.^20^ noticed that EVs from colorectal cancer cells interfere with differentiation of blood monocytes to macrophages, leading to differences in macrophage morphology and M1/M2 markers levels which depends on the time of the first contact between monocytes and EVs. However, it still needs to be investigated in which circumstances tumor-derived EVs can circumvent macrophages towards a pro-tumoral phenotype or induce an anti-tumoral activity. Based on this, we asked whether EVs secreted by melanoma cells under chemotherapy can induce a phenotype reprogramming in tumor cells and also in macrophages, affecting tumoral response to alkylating drugs hampering their efficacy. Our results indicate that murine and human melanoma cells secrete higher amount of EVs upon temozolomide (TMZ) and cisplatin (CDDP) treatment. These vesicles are taken up by tumor cells; however, they do not confer growth advantage and/or drug resistance to naïve melanoma cells *in vitro*, as demonstrated in other tumor types. On the other hand, EVs secreted by melanoma cells in response to TMZ push macrophages towards M2 phenotype. Moreover, when admixed with TMZ-treated cells and injected in nude mice, they promote the growth of the remaining viable tumor cells also through a nuclear reprogramming, constituting a novel route for tumor repopulation and treatment failure in melanomas.

## Results

### Extracellular vesicles (EVs) shedding by melanoma cells upon chemotherapy

**Figure 1:**
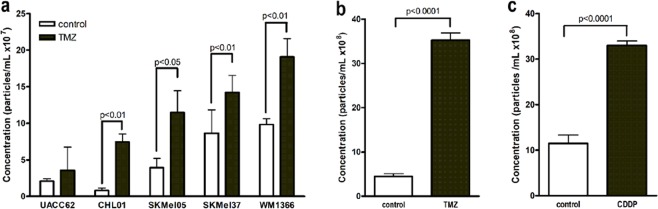
Chemotherapy increases extracellular vesicles (EVs) shedding by melanoma cells. Quantification of secreted EVs by Nanoparticle Tracking Analysis (NTA) in response to TMZ in human (**a**) and murine melanoma cells (**b**). (**c**) Quantification of EVs secreted after CDDP treatment in murine melanoma cells. Bars represent the mean of three measurements ± SD. Results were considered significant when p < 0.05 (n = 3–9) by unpaired t test (one-tailed).

Since EVs may constitute a novel adaptative strategy employed by tumor cells to overcome stressful conditions in tumor microenvironment, we evaluated whether chemotherapy modulates EVs shedding by melanoma cells. To address this point, we treated five human melanoma cell lines (UACC 62, CHL-01, SKMel 05, SKMel 37 and WM1366) with TMZ (360 µM) for 72 h and quantified the amount of EVs secreted under these conditions. As shown in Fig. 1a and Table 1, we observed a significant increase in EVs secreted by 4 out of 5 human melanoma cell lines under these conditions (p = 0.0073 for CHL01, p = 0.013 for SKMel05, p = 0.0032 for SKMel37 and p = 0.0085 for WM1366), which led us to hypothesize that these EVs might interfere with melanoma response to chemotherapy. We also observed an increase in EVs shedding by a murine melanoma cell line, B16F10, in response to TMZ (72 h; Fig. 1b; p < 0.0001), indicating that the release of EVs under a stress condition like chemotherapy is a common event in human and murine melanoma. In addition, to determine whether this response is specific to TMZ, we treated B16F10 cells with another chemotherapeutic drug commonly used in clinic to treat metastatic melanoma in low and middle income countries^21,22^, cisplatin (25 µM; 24 h), and we did observe a significant increase in the amount of EVs released by these cells (Fig. 1c; p < 0.0001), suggesting that alkylating drugs induce vesicle shedding by melanoma cells.

**Table 1:** Extracellular vesicles shedding by human melanoma cells in response to temozolomide.

Melanoma cell line	Control (×10^7^ particles/mL) Mean ± SD	TMZ (×10^7^ particles/mL) Mean ± SD	p value
UACC62	2.09 ± 0.31	3.55 ± 3.18	n.s.
CHL01	0.82 ± 0.31	7.42 ± 1.09	0.0073
SKMel-05	3.92 ± 1.27	11.48 ± 2.99	0.013
SKMel-37	8.63 ± 3.20	14.20 ± 2.34	0.0032
WM1366	9.83 ± 0.79	19.06 ± 2.50	0.0085

### Characterization of EVs released by melanoma cells in response to chemotherapy

**Figure 2:**
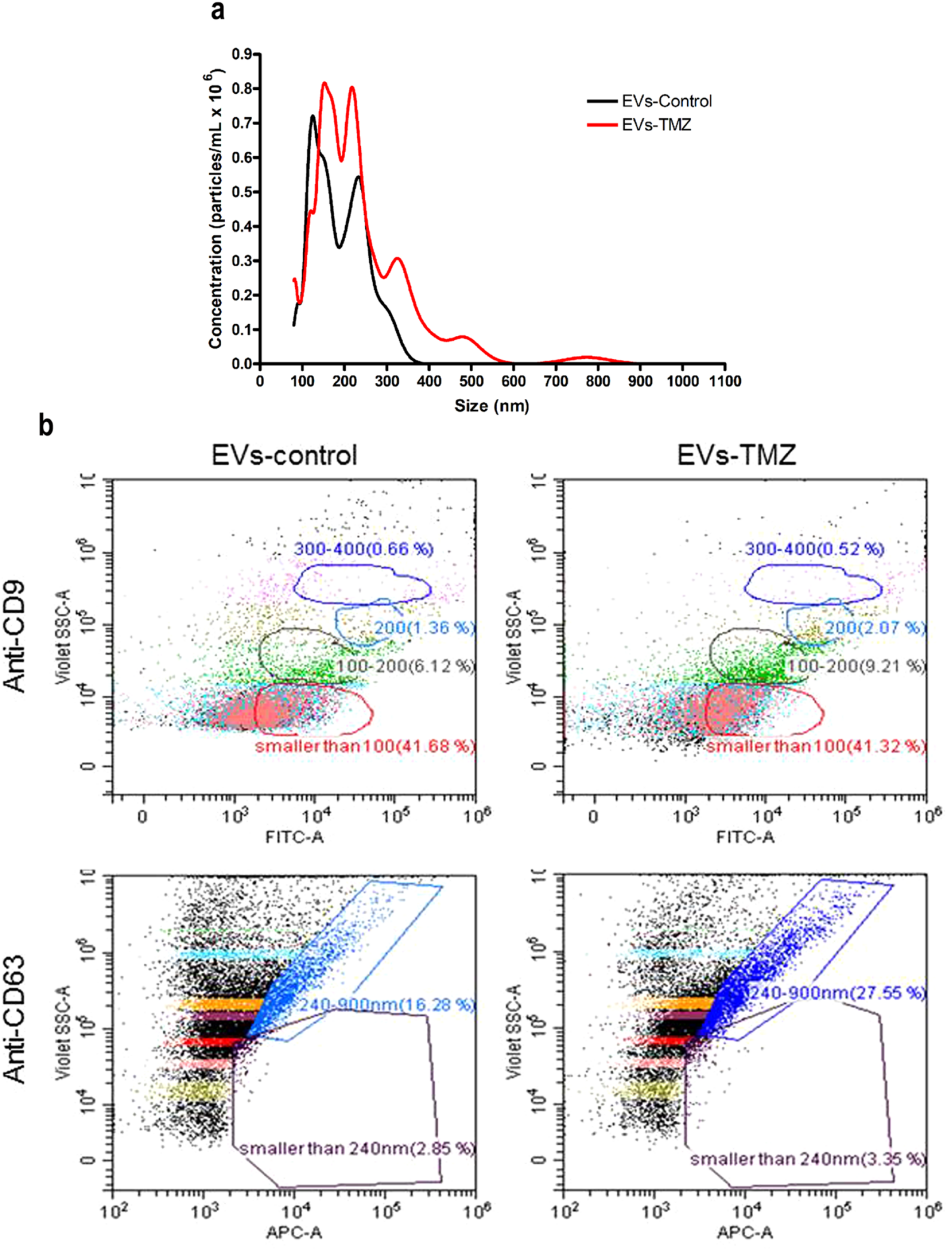
Characterization of EVs secreted by melanoma cells in response to TMZ. (**a**) A representative NTA showing the size and concentration distribution of EVs secreted by SKMel 37 human melanoma cells treated or not with TMZ. Each curve was generated from three measurements. The mean ± SD for control was 189 nm ± 14.7 and 237 nm ± 11.7 for TMZ-treated cells (p = 0.0116 by unpaired t test, two-tailed). (**b**) Dot plots of EVs from SKMel 37 cells showing the presence of the vesicle protein marker CD9 and CD63. The percentages of CD9 and CD63 positive events are shown inside the gates. Each gate represents a specific vesicle size range (<100 nm, 100–200 nm, 200 nm, 300–400 nm).

We next evaluated the size distribution profile of EVs secreted by human melanoma cells upon TMZ treatment by nanoparticle tracking analysis (NTA). As shown in Fig. 2a, the majority of secreted vesicles by SKMel 37 cells range from 100 to 350–400 nm in size, comprising both EVs entities (exosome and microvesicles). It is interesting to notice that the treatment with TMZ alter the size distribution profile of EVs (189 nm ± 14.7 and 237 nm ± 11.7 for control and TMZ, respectively, p = 0.0116), as observed by others in some stressful conditions like low pH^23^. EVs secreted under these conditions were also analyzed for the presence of the vesicle marker proteins, CD9 and CD63, using the Cytoflex flow cytometry. We used a mix with beads ranging in size from 100 nm to 1 µM to calibrate the equipment and in all tested cell lines, treated or not with TMZ, we detected CD9^+^ and CD63^+^ vesicles (Fig. 2b and Supplementary Fig. 1).

### EVs uptake does not modulate drug sensitivity in melanoma cells *in vitro*

**Figure 3:**
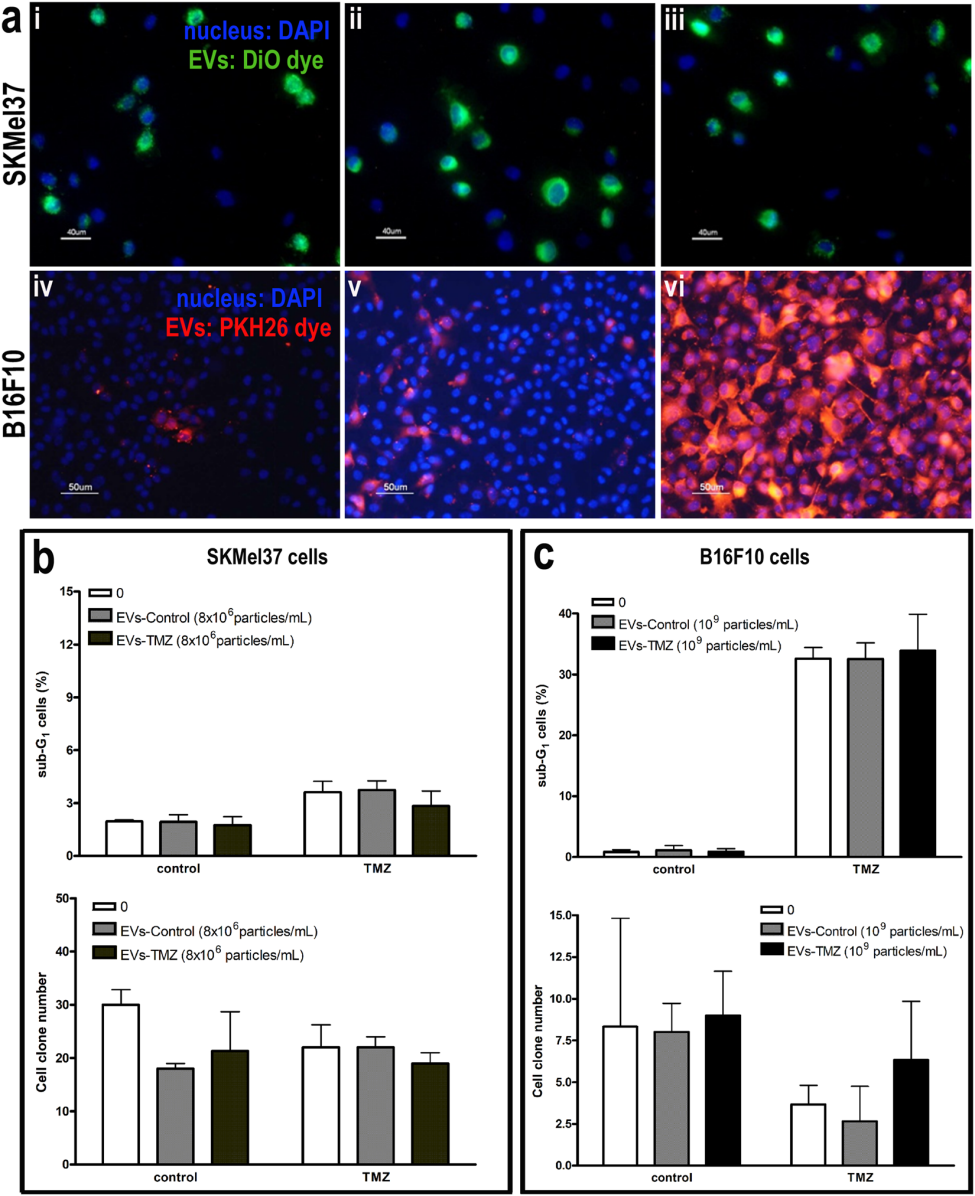
EVs shedding by melanoma cells after TMZ treatment do not modulate cell sensitivity to chemotherapy *in vitro*. In (**a**) EVs uptake by SKMel 37 (i,ii,iii) and B16F10 (iv,v,vi) melanoma cells. Scale bars, 50 and 40 µm. In (**b**) SKMel 37 and (**c**) B16F10 cells were treated with TMZ in the presence of EVs from pre-treated cells (DMSO or TMZ) and cell death was evaluated by flow cytometry after propidium iodide staining (upper graphs). Clonogenic assay was also performed for both cell lines (lower graphs) (n = 6–9). Bars indicate mean ± SD.

Since the release of EVs has been correlated with the acquisition of a chemoresistant phenotype by tumor cells, we next evaluated whether EVs secreted by melanoma cells in response to chemotherapy could modulate the drug response in these cells. We first evaluated EVs uptake by human and murine melanoma cells and, as shown in Fig. 3a, this uptake seemed to be dose dependent. Then, naïve SKMel37 human melanoma cells were treated with TMZ in the presence of EVs secreted by SKMel37 cells previously treated or not with this drug. After 72 h, SKMel37 sensitivity to TMZ was not altered by EVs, neither in response to acute drug exposure (sub-G1 cells) nor in long-term response (cell clone number) as shown in Fig. 3b. The lack of acute and long-term effects by EVs from treated cells in TMZ sensitivity was also observed in B16F10 cells (Fig. 3c). Regarding cisplatin, we also did not observe any differential response to this drug in B16F10 cells in the presence of EVs from pre-treated cells (Supplementary Fig. 2), indicating that EVs secreted by melanoma cells in response to acute treatment with chemotherapy do not confer resistance to naïve tumor cells *in vitro*.

### EVs shed by melanoma cells in response to TMZ induce macrophage polarization towards M2 phenotype

**Figure 4:**
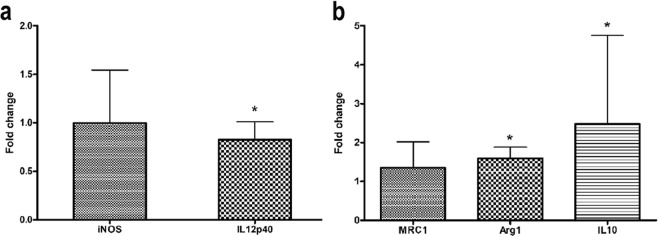
Extracellular vesicles (EVs) shedding by murine melanoma cells after TMZ treatment induces macrophage polarization towards M2 phenotype. qRT-PCR for analyzing the expression level of typical M1 (iNOS and IL12p40) (**a**) and M2 (MRC1, Arg1 and IL10) (**b**) genes in F4/80^+^ macrophages in the presence of EVs from control- and TMZ-treated cells. HPRT was used as housekeeping gene. EVs from control cells were used as the reference and EVs from TMZ-treated cells as the target sample. Bars indicate mean ± SD of five independent experiments. Results were considered significant when p < 0.05 by Mann-Whitney test.

It is well known that stromal cells in tumor microenvironment support tumor growth and modulate tumor response to therapy, which depends, at least in part, on tumor-stromal cell communication. One way to guarantee this orchestrated communication among both cell types relies on the uptake of EVs. Based on the fact that tumor macrophages are particularly abundant in many human malignancies promoting tumor progression and are professional phagocytic cells^24^, we decided to verify whether EVs shed by melanoma cells in response to chemotherapy might modulate macrophage phenotype. To test this hypothesis, we collected bone marrow cells from C57BL/6 mice femur and cultured them in RPMI plus L929 supernatant and FBS to obtain F4/80^+^ macrophages (Supplementary Fig. 3A). After that, macrophages were induced to M1 and M2 polarization in the presence of EVs secreted by B16F10 murine melanoma cells in response to TMZ treatment (360 µM for 72 h) or control (DMSO) (Supplementary Fig. 3B). After 24 h gene expression analyses were carried out to verify whether EVs could influence this phenotype switching. Interestingly, we noticed a significant increase in M2-related gene expression, Arg-1 and IL-10, in M2 macrophages in the presence of EVs from TMZ-treated cells (p = 0.0022 and p = 0.04, respectively). Regarding M1-related genes, the same EVs caused a significant reduction in IL12p40 expression in macrophages polarized to M1 (p = 0.04; Fig. 4), suggesting that EVs derived from TMZ-melanoma treated cells induce a pro-tumoral phenotype in macrophages that might contribute to melanoma progression.

### EVs shed by melanoma cells in response to TMZ favor melanoma growth *in vivo*

**Figure 5:**
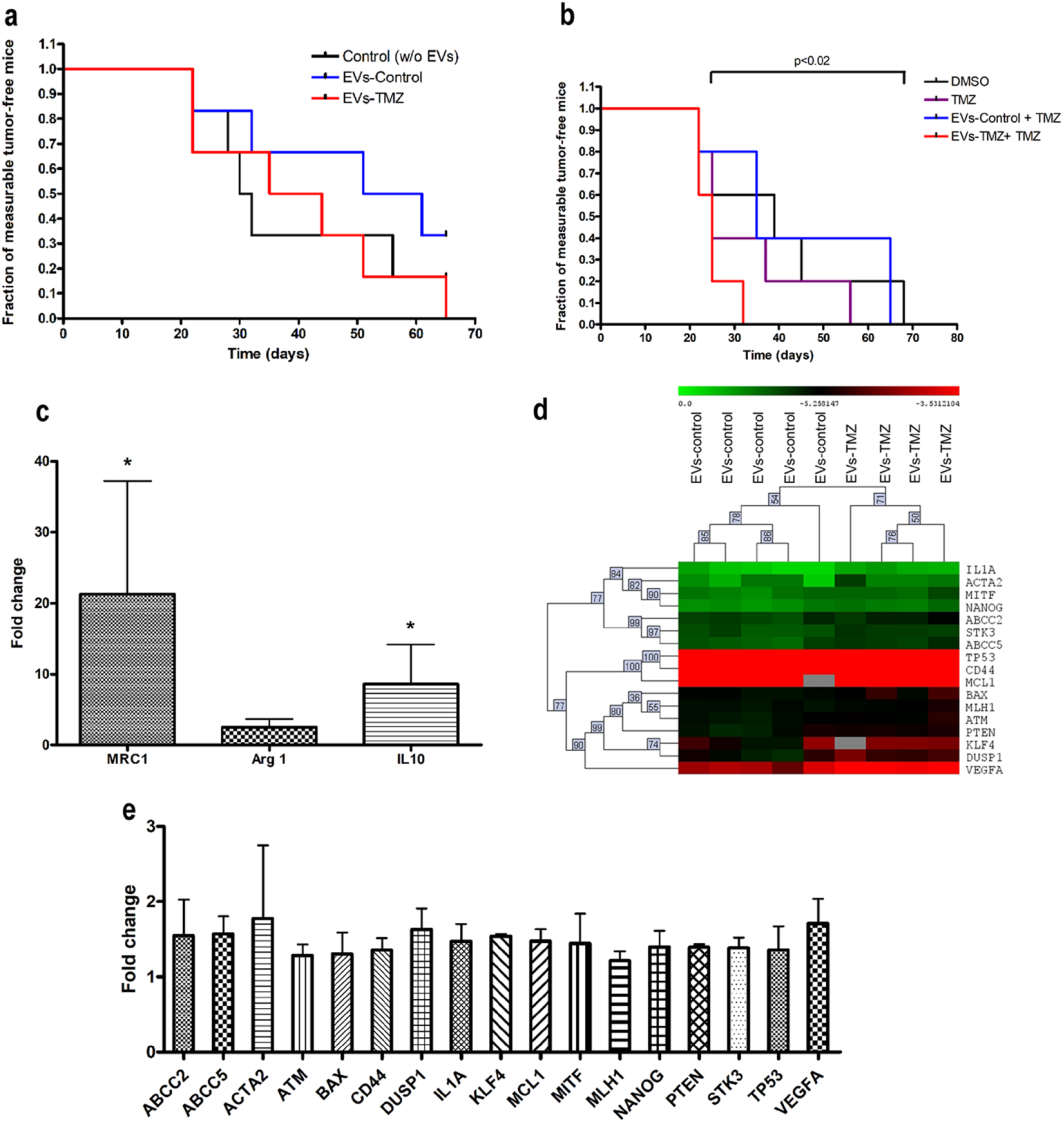
Extracellular vesicles (EVs) shedding by human melanoma cells after TMZ treatment favor tumor growth *in vivo*. In (**a**) SKMel37 cells were injected s.c. into male nude mice admixed or not with EVs from control or TMZ-treated cells (control- and TMZ-EVs, respectively) in male nude mice (n = 6). In (**b**) animals were injected s.c. with SKMel37 pre-treated or not with TMZ in the presence of control- or TMZ-EVs (n = 5). Results were considered significant when p < 0.05 by Logrank test. (**c**) qRT-PCR analysis showing the fold change in M2 gene expression in tumors derived from SKMel37 pre-treated with TMZ in the presence of control or TMZ-derived EVs (n = 4). Bars indicate mean ± SD. HPRT was used as housekeeping gene. Results were considered significant when p < 0.05 by Mann-Whitney test. (**d**) Heat map showing hierarchical clustering of 17 differentially expressed genes in tumors derived from human SKMel37 pre-treated cells with TMZ in the presence of control- or TMZ-EVs. Data were analyzed by MeV software (200 permutations; FDR 2.89%). (**e**) Microfluidic-based qRT-PCR showing the fold change in the 17 genes differentially expressed in tumors from SKMel37 pre-treated cells admixed with TMZ-EVs. Bars indicate mean ± SD. RPLPO was used as housekeeping gene.

Based on our findings, we next postulated that EVs release by melanoma cells in response to TMZ could favor melanoma repopulation after chemotherapy through modulation of macrophage polarization in tumor microenvironment. To evaluate this, we used SKMel37 human cells because the engraftment and growth of this cell line in nude mice is more efficient and reproducible than the others. Then, SKMel37 cells were treated or not with TMZ in the presence of EVs secreted by cells previously treated with this drug. Then, cells were harvested and injected (5 × 10^5^/animal) s.c. in Balb/C nude mice admixed with TMZ- or control-derived vesicles (TMZ-EVs and control-EVs, respectively). We did not observe a significant difference when animals were injected with SKMel 37 cells admixed with TMZ-EVs in comparison to control (Fig. 5a). However, when animals were injected with TMZ-treated cells mixed with TMZ-EVs, we observed a remarkable decrease in the fraction of measurable tumor free mice compared to animals injected with SKMel 37 treated cells admixed with control-EVs (p = 0.0228) (Fig. 5b), indicating that under chemotherapy EVs secretion could promote the outgrowth of resistant cells. Since TMZ-EVs modulate macrophage polarization towards M2 phenotype, we speculated that this tumor promoting effect could be, at least in part, due to an exacerbated M2 macrophage polarization during chemotherapy favoring tumor repopulation. To address this point, tumors xenografts were excised when reached 300–400 mm^3^ in size and M2 marker genes were evaluated by real time PCR. All reactions were carried out using primers against mouse genes in order to assess the gene expression levels on stromal cells such as macrophages. As shown in Fig. 5c, we found a strikingly increased expression of the M2-related markers, MRC1 and IL10, in tumors originated from TMZ-treated cells admixed with TMZ-EVs in comparison to control (p = 0.0286 for both genes), suggesting that EVs secreted by tumor cells during chemotherapy promote tumor repopulation possibly by acting in tumor macrophages inducing a pro-tumoral phenotype. Moreover, in order to verify if TMZ-EVs also induce a nuclear reprogramming in tumor cells during repopulation, the expression levels of 96 human genes were measured in these xenografts using Fluidigm Biomark system. As observed in Fig. 5d, 17 genes were found to be significantly differentially expressed between tumors derived from TMZ-treated cells admixed with control and TMZ –EVs (FDR = 2.89%). Of note, all 17 genes were up-regulated in animals inoculated with TMZ-EVs in comparison to control group (control-EVs) (Fig. 5e), including ATM (ATM serine/threonine kinase), MLH-1 (mutL homolog 1), MCL1 (BCL2 family apoptosis regulator) and MITF (Microphthalmia-Associated Transcription Factor). This set of genes are involved in DNA damage response, DNA repair and melanoma cell survival and probably play an important role in melanoma regrowth after TMZ treatment. Interestingly, we also observed an up-regulation in NANOG (Nanog homeobox), ABCC2 and 5 (ATP binding cassette subfamily C member 2 and 5), CD44 (CD44 molecule (Indian blood group) and KLF4 (Kruppel like factor 4), all cancer stem cells related genes, which suggests that the acquisition of a stemness phenotype by melanoma cells promotes cell survival under chemotherapy. In addition, VEGF-A (Vascular Endothelial Growth Factor-A), a pro-angiogenic factor crucial for tumor growth and progression, showed increased levels in TMZ-EVs group. These findings corroborate our hypothesis that secreted EVs in response to chemotherapy promote melanoma repopulation inducing a cellular reprogramming in both macrophages and tumor cells.

## Discussion

EVs have been described as important mediators of cell communication in tumor microenvironment, contributing to tumor progression and metastasis. Tumor-derived EVs promote angiogenesis^25,26^, suppression of immune system^27–29^, metastasis establishment^16,17^ and extracellular matrix remodeling^30^, dictating tumor natural history. Several reports have shown that normal and tumor cells vesiculate in response to different stresses like hypoxia^31–33^, radiation^31^, acidosis^34^ and cytotoxic drugs^35^, modifying cell signaling pathways and phenotype in the recipient cell over short and long distances. Based on that, we asked whether the stress induced by chemotherapy in melanoma modulates the production and secretion of EVs by tumor cells which could alter cell communication within tumor microenvironment. We did find a significant increase in EVs shedding by both human and murine melanoma cell lines in response to TMZ and CDDP, chemotherapeutic drugs commonly used in clinic for metastatic melanoma, indicating that increased levels of EVs might constitute a biomarker of melanoma behavior during chemotherapy.

In fact, it is largely accepted that therapeutic intervention may constitute a selective pressure for arising and expansion of resistant tumor cells according to Darwinian natural selection rules. In this context, EVs have been recently implicated as important mediators in adaptive mechanisms by tumor cells in stressful conditions^3^. Recent findings have been demonstrated that these EVs are responsible for chemoresistance in many tumor types. Xiao *et al*.^11^ showed that exosomes derived from lung cancer cells under cisplatin treatment increased the resistance of these cells to this compound. In breast cancer cells, drug resistance is modulated by glycoprotein-P delivery by exosomes^36^. Sunitinib resistance in renal carcinoma is also transmitted by horizontal transfer of lncARSR, a long non coding RNA, via exosomes^37^. Recently, it has also been shown that EVs are involved in the bystander effect following cisplatin treatment in ovarian carcinoma^38^. The EVs secreted by these cells in response to cisplatin induced drug resistance in naïve tumor cells and the blockage of EVs uptake abrogates the acquisition of drug resistance. In melanoma harboring BRAF V600E mutation, the resistance to vemurafenib is conferred to sensitive cells by PDGFRβ transfer through EVs, leading to treatment failure^39^. Based on these studies, we postulated that EVs secreted by melanoma cells in response to TMZ and CDDP could confer a growth advantage and drug resistance to naïve tumor cells. We observed that both human and murine melanoma cells engulfed EVs secreted by their own in a dose dependent manner, as reported in a recent study by Matsumoto *et al*.^40^. Using a murine melanoma model, they verified that the uptake of their own exosomes by B16BL6 cells led to an increase in cell proliferation rate accompanied by a decrease in apoptotic basal levels promoting tumor progression. However, in our study, EVs from murine and human melanoma cells did not confer any growth advantage for melanoma cells *in vitro*. Regarding the EVs derived from TMZ or cisplatin-treated cells, we also did not to notice any modification in the sensitivity of naïve cells to these drugs *in vitro*.

Since tumor progression is dictated by tumor-stromal cell interaction, we next sought to determine whether EVs derived from TMZ-treated cells are involved in this network. Tumor-associated macrophages are among the most abundant non-transformed cells in tumor microenvironment and are educated by tumor cells to promote its progression^24^. Chang *et al*.^41^ recently showed that colon carcinoma cell debris generated in response to chemotherapy with 5-FU are able to induce tumor outgrowth through osteopontin secretion by tumor cells and macrophages. Another way to educate these stromal cells is through the engulfment of EVs secreted by malignant cells followed by phenotype reprogramming that can favor or impair tumor growth and progression. In this context, we found that under chemotherapy, tumor cells can induce macrophage polarization towards M2 phenotype by secreted EVs. In the presence of EVs derived from murine melanoma cells treated with TMZ, we observed an upregulation in M2-related genes and a decrease in IL12p40, a known M1 marker, in bone marrow derived-macrophages. de Vrij *et al*.^42^ showed that glioblastoma-derived EVs induced a higher expression of M2-markers in monocyte-derived macrophages which was accompanied by higher phagocytic capacity of these cells. In gastric cancer, it was demonstrated that exosomes derived from tumor cells also promoted macrophage polarization towards to M2 phenotype, leading to an increase in tumor cell migration and invasion^19^. In colorectal cancer, Shinohara *et al*.^43^ noticed that EVs derived from tumor cells promoted THP-1 polarization to M2 which was dependent on miR-145 transfer through exosomes. The authors also demonstrated that co-injection of tumor cells and macrophages polarized with EVs promoted tumor growth in nude mice. As demonstrated in this study, Piao *et al*.^44^ also found increased levels of arginase and CD206 in macrophages incubated with exosomes from breast cancer cells *in vitro* and *in vivo*, evidencing the role of EVs in tumor cell and macrophage communication within tumor microenvironment.

Conversely, others studies reported that tumor-derived EVs can induce macrophage polarization towards both M1 and M2 which seems to be dependent on vesicle cargo and cell context^20^. In melanoma, it was reported that exosomes derived from B16F10 murine melanoma cells stimulated the production of both M1 and M2 cytokines by primary macrophages and RAW 264.7 macrophage cell line^45^. Then in order to elucidate the role of EVs derived from TMZ-treated melanoma cells in melanoma progression, we conducted an *in vivo* experiment and showed that EVs derived from human melanoma cells under TMZ treatment indeed favor tumor growth in animals injected with cells previously treated with this drug, suggesting that EVs could constitute a route for tumor repopulation after chemotherapy with alkylating compounds in melanoma. Since we also observed an up-regulation of M2 gene expression in tumor xenografts from this group, we might speculate that this route involves the polarization of macrophages towards the pro-tumoral phenotype by TMZ-derived EVs. Although we did not observe any phenotypic change caused by EVs in tumor cells *in vitro*, we found that EVs derived from TMZ-treated cells indeed promoted a genetic reprogramming in melanoma cells *in vivo*. This cellular reprogramming was characterized by an up-regulation of ATM, MLH1, MCL1 and MITF, genes known to be involved in DNA repair, cell survival and proliferation - essential pathways for tumor survival under stressful conditions like chemotherapy^46,47^. In fact, overexpression of the anti-apoptotic gene MCL-1, as well as the transcription factor MITF, have been described as important modulators of chemoresistance in melanomas^48,49^. Based on this, we postulated that EVs secreted under TMZ treatment induce chemoresistance *in vivo* through up-regulation of genes involved in DNA damage response, repair and cell survival, promoting tumor regrowth after treatment. Additionally, we also observed an up-regulation in some cancer stem cell markers such as NANOG, ABCC2, ABCC5, CD44 and KLF4^50,51^. In fact, resistance to chemo- and radiotherapy have been attributed to tumor cells that acquire some properties of embryonic/pluripotent stem cells, including the expression of stem cells markers and the ability to self-renew^52,53^. Since the so-called cancer stem cells are thought to be responsible for tumor resistance in many solid tumors including melanoma^54,55^, it is reasonable to assume that tumor repopulation after chemotherapy is mediated by secreted EVs that induce a stemness signature in the recipient viable tumor cells promoting tumor re-growth and progression.

Moreover, we found that EVs secreted under these conditions caused an increase in VEGF-A gene expression in tumor cells. As tumor angiogenesis is a *sine qua non* condition for tumor progression, this up-regulation may result in sustained angiogenesis during melanoma re-growth. Furthermore, we noticed an increase in DUSP1 (Dual Specificity Phosphatase 1), STK3 (Serine/Threonine Kinase 3), ACTA2 (Actin, alpha 2, smooth muscle, aorta) and IL1A (Interleukin 1α) gene expression levels; however, the role of these genes in melanoma repopulation remains to be addressed. Furthermore, the role of EVs released in response to chemotherapy dictating tumor progression was also observed recently by Keklikoglou *et al*.^56^. Using a breast cancer model, the authors showed that chemotherapy-elicited EVs secreted by tumor cells induced pulmonary CCl2 expression, LyC6+ expansion and endothelial cell activation in lung, promoting the establishment of metastasis. These findings reinforce our hypothesis that EVs secreted by tumor cells under chemotherapy can be responsible for treatment failure.

In summary, our study shows that chemotherapy with alkylating drugs induces the secretion of exosomes and microvesicles by melanoma cells. The injection of TMZ-treated melanoma cells admixed with EVs from previously treated cells boosted melanoma outgrowth through up-regulation of genes related to cell survival, DNA repair and stemness, indicating that these vesicles prompt melanoma growth after chemotherapy contributing to treatment failure. Using a murine model, we found evidence that these EVs can be taken up by macrophages skewing their activation towards the M2 phenotype. This finding suggests that stressed tumor cells due to chemotherapeutic challenge continue to interact with stromal cells such as macrophages establishing new routes for tumor repopulation via EVs. Future studies will determine whether these EVs can also reprogram macrophages in human tumors.

## Methods

### Cell culture

Human melanoma cell lines were cultivated in DMEM (CHL01, SKMel05, UACC62 and WM1366) or MEM (SKMel37) and B16F10 murine melanoma cells in RPMI1610 medium supplemented with 10% fetal bovine serum (FBS; ThermoFisher Scientific, Waltham, MA). All cells lines were tested for mycoplasm routinely.

### Extracellular vesicles (EVs) depletion from Fetal Bovine Serum (FBS)

In order to exclude EVs present in serum, FBS were ultracentrifuged (Beckman, California, USA) at 100,000 g for 16 h at 4 °C.

### EVs isolation and quantification

For EVs isolation, cell culture media were consecutively centrifuged at 300 g for 10 min, at 2,000 g for 15 min and at 10,000 g for 30 min at 4 °C. Then, the supernatant was ultracentrifuged at 100,000 g for 2 h. The pellet enriched in EVs was washed once with PBS. For particle size distribution and concentration quantification, samples were diluted in PBS and loaded into NanoSight LM10 (Malvern, UK) using a syringe. Nanoparticle movement was captured for 60 s at 25 °C 3 times. Recorded videos were subjected to Nanoparticle Tracking Analysis (NTA) Software and size distribution and concentration were determined.

### EVs characterization by flow cytometry

10^7^ EVs derived from each cell line were diluted in PBS in a final volume of 50 µL. Anti-human CD9 (1:100; Cymbus Biotechnology, London, UK) and anti-human CD63 (1:100; clone H5C6, BD Pharmingen, California, USA) were added and samples were incubated for 1 h at RT and, after that, anti-mouse IgG FITC or anti-mouse Alexa 647 (1:200; ThermoFisher Scientific, Waltham, MA) were added and incubation proceeded for 1 h at RT. For staining control, EVs were incubated only with secondary antibody. 1 mL of filtered PBS was added to each sample and the fluorescent intensity were determined using CytoFLEX flow cytometry (Beckman Coulter, CA, USA). The equipment was calibrated using a mixture of fluorescent beads with size ranging from 100 nm to 1 µm for vesicles detection. Data analysis was performed with CytExpert 2.0 Software.

### EVs staining and uptake assay

For EVs uptake experiments, EVs were stained with PKH26 (Sigma, St. Louis, MO) or DiO (ThermoFisher Scientific, Waltham, MA) according to manufacturer’s recommendations. B16F10 or SKMel 37 cells were incubated O.N. with crescent concentrations of stained EVs. Nuclei were stained with Hoescht33342 (25 µg/mL) and images were collected in a fluorescence microscope (Nikon).

### EVs and chemotherapeutic drug treatment

SKMel37 or B16F10 cells were plated with media containing 10% of FBS depleted of EVs. On the following day, 8 × 10^6^ or 10^9^ EVs/mL from previously treated or not treated human or murine melanoma cells were added to the cells with or without TMZ (360 µM, 72 hs) or CDDP (25 µM, 24 hs; Sigma, St Louis, MO). EVs were added daily and, after 24 or 72 h, cell number and death were determined.

### Cell death assays

Cells were fixed with 70% ethanol and incubated for at least 2 h at RT and then incubated in a solution containing 20 µg/mL propidium iodide, 5 µg/mL RNase A (both ThermoFisher Scientific, Waltham, MA) and 1% Triton X-100 (Sigma, St Louis, MO) for 30 min. Cell cycle analysis and sub-G1 population were evaluated in Attune flow cytometry (ThermoFisher Scientific, Waltham, MA). For clonogenic assays, after TMZ or CDDP treatment, cells were plated at low density and maintained in cell culture for 7–10 days or until visible colony formation. Then, cells were fixed with 1% formaldehyde for 15 min, stained with 1% violet crystal and visible clones were counted.

### Macrophage polarization studies

L929 cells were maintained in DMEM supplemented with 10% FBS and 50 µM 2-mercaptoethanol for 4–5 days. Supernatant was collected and filtered (0.22 µm) prior to utilization. Bone marrow cells from C57BL/6 mice (8 weeks, male) femur were collected and cultivated in RPMI1640 supplemented with 30% L929-conditioned medium and 15% FBS during 6 days. 10^6^ cells were incubated with Fc-block (rat anti-mouse CD16/CD32; 1 µg; BD, San Jose, CA) for 1 h on ice. After that, cells were incubated with F4/80 antibody conjugated to PE (0.25 µg; BD, San Jose, CA) or isotype control (anti-rat-IgG2a; 0.25 µg, ThermoFisher Scientific, Waltham, MA) for 45 min on ice. Immunostaining of F4/80 positive cells were determined by flow cytometry. F4/80^+^ macrophages were cultured in RPMI media containing 10% of FBS, LPS (1 µg/mL; Sigma, St Louis, MO) and IFN-γ (50 ng/mL; R&D Systems, Minneapolis, MN) or IL-4 (50 ng/mL; R&D Systems, Minneapolis, MN) in the presence of EVs (10^9^ EVs/mL) derived from TMZ or vehicle (DMSO) B16F10 treated cells to induce M1 or M2 polarization, respectively. After 24hs, RNA was collected using Trizol® (ThermoFisher Scientific, Waltham, MA) following manufacturer’s instructions. cDNA was synthesized from 2 µg of RNA using High Capacity kit (ThermoFisher Scientific, Waltham, MA) and real-time PCR was performed using SyBR Green (SYBR Green Master Mix; Thermo Fisher Scientific, Waltham, MA) chemistry. qRT-PCR was carried out for the following mouse genes: IL-12 (Forward: 5′-AGCAGTAGCAGTTCCCCTGA-3′; Reverse: 5′-AGTCCCTTTGGTCCAGTGTG-3′), iNOS (Forward 5′-CAGGAACCTACCAGCTCACTCT-3′; Reverse 5′-ATGTGCTGAAACATTTCCTGTG-3′), MRC1 (Forward 5′-TTTGCAAGCTTGTAGGAAGGA-3′; Reverse 5′-CCAATCCACAGCTCATCATTT-3′), Arginase-1 (Forward 5′-GAACCCAACTCTTGGGAAGAC-3′; Reverse 5′-GGAGAAGGCGTTTGCTTAGTT-3′) and IL-10 (Forward 5′-ACTTGCTCTTGCACTACCAAAGCC-3′; Reverse 5′-GCATGTGGCTCTGGCCGACTG-3′) and HPRT as endogenous control (Forward 5′-AGGCCAGACTTTGTTGGATTT-3′; Reverse 5′-GGCTTTGTATTTGGCTTTTCC-3′). The relative gene expression (fold change) was obtained by the 2^-delta deltaCt method, according to Livak and Schmittgen^57^, using the EVs from control group as the reference and EVs from TMZ-treated cells as the target sample. The reaction was performed in ABI 7500 Real Time PCR machine (Thermo Fisher Scientific, Waltham, MA).

### Tumor growth *in vivo*

SKMel37 human melanoma cells were treated or not with 360 uM TMZ for 72 h in the presence of EVs (10^6^ EVs/mL) derived from SKMel37 previously treated with TMZ or vehicle. After that, 5 × 10^5^ cells admixed with EVs from TMZ or vehicle-treated cells were injected s.c. in male Balb/C nude (8 weeks old). Animals were monitored daily. For gene expression analysis, animals were euthanized when tumors reached 300–400 mm^3^ in volume. Tumors were excised and dissociated with a tissue homogenizer (Brinkman Instruments, NY, USA) and RNA was extracted using Trizol®. As described above, cDNA was synthesized from 2 µg of RNA using the High Capacity kit and real-time PCR was performed using SyBR Green. M2-gene expression was determined using the mouse primers listed above. All animal experiments were conducted according to legislation for animal research in Brazil with approval from the Committee on Ethics of Animal Experiments of the University of São Paulo, Faculty of Medicine, CEP-FMUSP, process 052/17. Animals were maintained under 12:12 light-dark cycle with food and water available *ad libitm*.

### Gene Expression Analysis Using EvaGreen^®^ on the Fluidigm BioMark HD System

A total of 14 ng of total RNA was used as initial input for cDNA synthesis (*Reverse Transcription master mix*, Fluidigm, California, USA) followed by 10 cycles of a Preamplification reaction (*Preamp Master Mix*, Fluidigm), according to manufacturer’s instructions. Preamplification was performed using a pool containing all the ninety six targets of interest, using a final concentration of 500 nM for each Delta Gene™ assay. We analyzed eighty nine target genes and seven endogenous genes. The assays contained Forward and Reverse Primers and were designed using D3 Assay Design software (Fluidigm). Supplementary Table 1 shows the complete information regarding all the ninety six Delta Gene™ assays under investigation. Preamplified cDNA was treated with 8U of Exonuclease I (New England BioLabs, MA, USA) to eliminate the carryover of unincorporated primers and the final product was diluted 10 times prior to qPCR. Then, we prepared a Sample mix solution containing the diluted pre-amplified cDNA product with SsoFast™ EvaGreen® Supermix with Low ROX (Bio-Rad Laboratories, CA, USA), according to manufacturer instructions. Each one of the 96 Delta Gene™ Assays (at a final concentration of 5 uM) and each Sample mix were loaded into the 96.96 Dynamic Array IFC (Integrated Fluidic Circuit) using the Juno IFC controller (Fluidigm).

qPCR was performed across multiple reaction chambers in nanoliter volume aliquots in the Fluidigm BioMark™ HD System Real-Time PCR according to GE Fast 96 × 96 PCR + Melt v2.pcl protocol. Data were collected by Biomark Data Collection software version 4.5.1 and were analyzed using the Fluidigm Real-Time PCR Analysis software version 4.3.1. We independently calculated a threshold value for each target gene and a cut-off of Ct = 22 was set as limit of detection. Using the NormFinder software, RPLPO gene showed to present the higher expression stability and was selected as the optimal normalization gene among the set of seven candidates for the calculation of the ∆Ct values.

### Statistical analysis

Unpaired t test and Logrank Test were performed using GraphPad Prism (San Diego, CA, USA). Statistical differences were considered when p ≤ 0.05. For macrophage gene expression, statistical comparisons of −∆Ct values between EVs-TMZ and control-EVs were performed using Mann-Whitney test in SPSS® 18.0 (SPSS, Inc, USA). The null hypothesis was rejected at p < 0.05. Fold Change values of TMZ-EVs group were calculated according to the comparative CT method (2−∆∆CT) using control-EVs group as reference. Histogram graphs and error bars represent the Fold Change and Standard deviation values, respectively. For EvaGreen Gene Expression (Fluidigm), −∆Ct values were loaded into MeV (Multiple Experiment Viewer) software version 4.9 (available at http://mev.tm4.org) for statistical analyses and hierarchical clustering construction. Data were compared using SAM (Significance Analysis of Microarrays) test (200 permutations; FDR 2.89%). The 17 differentially expressed genes (DEGs) were used to construct a Hierarchical clustering HCL, based on Euclidean Distance (Complete linkage). Fold change values of TMZ-EVs group were calculated according to 2−∆∆Ct formula using control-EVs as the reference group.

## Supplementary information

Supplementary Figures
