# Supplementary material for: Extracellular Vesicles Shedding Promotes Melanoma Growth in Response to Chemotherapy
Source: Sci Rep. 2019 Oct 9;9:14482. doi: 10.1038/s41598-019-50848-z (PMC6785560; doi:10.1038/s41598-019-50848-z)
Supplement: Supplementary file 1 — Supplementary Figures [file 41598_2019_50848_MOESM1_ESM.pdf]

EXTRACELLULAR VESICLES SHEDDING PROMOTES MELANOMA GROWTH IN RESPONSE TO CHEMOTHERAPY

Luciana Nogueira de Sousa Andrade*[§], Andréia Hanada Otake*[§], Silvia Guedes Braga Cardim, Felipe Ilelis da Silva, Mariana Mari Ikoma Sakamoto, Tatiane Katsue Furuya, Miyuki Uno, Fátima Solange Pasini and Roger Chammas.

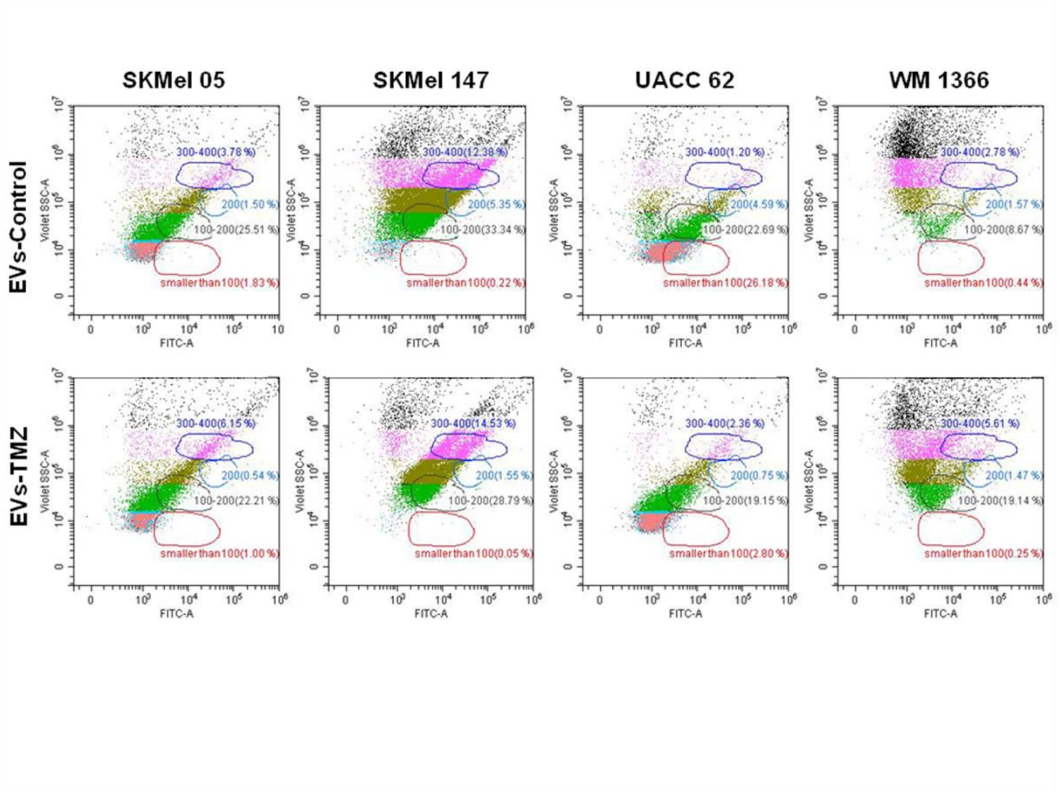

Supplementary Figure 1. Characterization of EVs secreted by human melanoma cells in response to TMZ. Dot plot of EVs from a panel of human melanoma cells showing the presence of CD9 positive vesicles secreted by all cell lines under normal condition and after TMZ treatment. The percentages of CD9 positive events are shown inside the gates. Each gate represents a specific vesicle size range (< 100nm, 100-200nm, 200nm, 300-400nm).

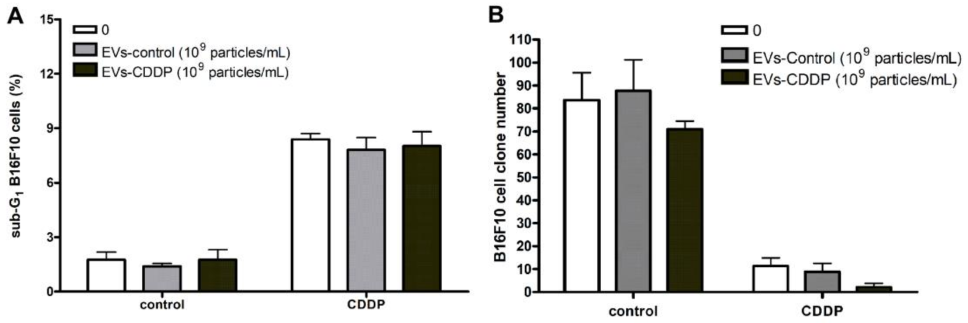

Supplementary Figure 2. Extracellular vesicles (EVs) shedding by murine melanoma cells after CDDP treatment does not modulate cell death. B16F10 cells were treated with cisplatin in the presence of EVs from pre-treated cells and in (A) cell death was evaluated by flow cytometry after propidium iodide staining (n=6). In (B) clonogenic assay was also performed (n=6). Bars indicate mean ± SD.

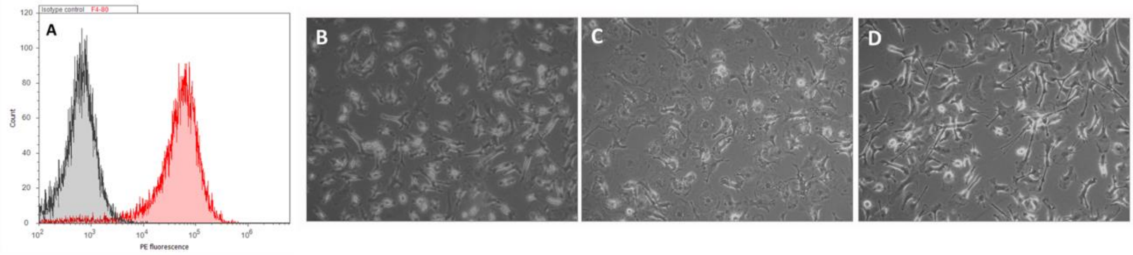

Supplementary Figure 3. Macrophage isolation from bone marrow cells. Bone marrow cells were collected from C57BL/6 mice femur and kept in cell culture in RPMI medium and L929 cells supernatant for 6 days. (**A**) Macrophage differentiation was evaluated by flow cytometer using F4/80 antibody. Morphological changes in F4/80$^+$ macrophages after incubation with (**B**) LPS (1μg/mL) and IFN-γ (50ng/mL) (**C**) or IL-4 (50ng/mL) (**D**) for M1 and M2 polarization, respectively. Magnification 10X.
